# Selection of the Maximum Spatial Cluster Size of the Spatial Scan Statistic by Using the Maximum Clustering Set-Proportion Statistic

**DOI:** 10.1371/journal.pone.0147918

**Published:** 2016-01-28

**Authors:** Yue Ma, Fei Yin, Tao Zhang, Xiaohua Andrew Zhou, Xiaosong Li

**Affiliations:** 1 West China School of Public Health, Sichuan University, Chengdu 610041, China; 2 Department of Biostatistics, School of Public Health, University of Washington, Seattle 98195, United States of America; Indiana University Bloomington, UNITED STATES

## Abstract

Spatial scan statistics are widely used in various fields. The performance of these statistics is influenced by parameters, such as maximum spatial cluster size, and can be improved by parameter selection using performance measures. Current performance measures are based on the presence of clusters and are thus inapplicable to data sets without known clusters. In this work, we propose a novel overall performance measure called maximum clustering set–proportion (MCS-P), which is based on the likelihood of the union of detected clusters and the applied dataset. MCS-P was compared with existing performance measures in a simulation study to select the maximum spatial cluster size. Results of other performance measures, such as sensitivity and misclassification, suggest that the spatial scan statistic achieves accurate results in most scenarios with the maximum spatial cluster sizes selected using MCS-P. Given that previously known clusters are not required in the proposed strategy, selection of the optimal maximum cluster size with MCS-P can improve the performance of the scan statistic in applications without identified clusters.

## Introduction

Spatial scan statistic, which was introduced by Kulldorff[1], focuses on detecting the presence and locations of geographic clusters within spatial datasets. The free software SaTScan[2] allows users to apply this statistic in different fields. A list of studies that utilized spatial scan statistic is posted in the SaTScan official website[3].

The maximum spatial cluster size is the only parameter that must be selected by users to apply commonly used circular spatial scan statistics with SaTScan software. This parameter is the maximum size that the scanning window can reach as scaled in terms of spatial distance or percentage of the total population at risk[4]. Ribeiro and Costa[5] investigated the performance of spatial scan statistics with different maximum spatial cluster sizes, including secondary clusters; they suggested that three performance measures are sensitive to the maximum spatial cluster size. Although simulation datasets support the selection of different maximum spatial cluster sizes for a specific cluster model, identifying a cluster model applicable for complex real datasets is complicated. Therefore, the guidelines for selecting the maximum spatial cluster sizes for real data remain unclear.

Kulldorff[6] reported that a window sized up to 50% of the population at risk can generally reduce negative clusters. Other researchers also selected lower values for practical reasons, such as data availability[7], location discontinuity[8], specific interest on small clusters[9], search for small clusters with high relative risk (RR)[10], low infectivity of a specific pathogen[11, 12], exploratory analysis for irregular-shaped clusters[8, 13], or limited available resources for intervention[5]. As such, simple selection of the maximum spatial cluster size may not be appropriate. The performance of the spatial scan statistic must be ranked with different parameters in an application because of varied relationship between the maximum spatial cluster size and the performance in different data sets. Therefore, a performance measure that is generally applicable for various applications must be used.

Numerous performance measures are commonly used in simulation studies; however, few of these measures can be easily applied in real data [14] because they are based on the presence of given artificial clusters. Identification of all the detected clusters as true or otherwise is usually impractical. For example, disease surveillance studies usually have limited available resources; moreover, several performance measures represent different aspects of performance[15]. As such, outcomes from multiple measures can be problematic when ranking the performance of different implementations of spatial scan statistics. Performance measures can be combined using specific formulas with arbitrary weights, but parameter selection is inevitably arbitrary. If the overall performance, rather than a specific aspect of performance, is of interest, then the overall performance measure that is not based on the given artificial clusters would be less arbitrary.

Performance measures at the aggregation level are commonly used over data sets generated with a similar underlying model because the former can detect slight differences among spatial scan statistics with different parameters. However, these datasets do not exist in reality. Although simulation datasets can be generated from historical data by using clustering models, this approach is difficult especially when no such historical data exist[16]. In this regard, a performance measure for a single data set is preferred than a performance measure based on a batch of data sets generated with the same model.

In summary, an overall performance measure based on applied dataset, rather than the known presence of true clusters, can be used to select the optimal spatial parameters for improving the performance of spatial scan statistics in applications. However, to the best of our knowledge, this measure has not been developed yet.

This study proposes a novel overall performance measure, namely, maximum clustering set–proportion (MCS-P), which is based on the likelihood function and is customized for all significant clusters and applied dataset. A full definition of MCS-P and additional details are provided in the next section. This new performance measure is applicable to data sets without known clusters because the presence of clusters is unnecessary. Section 3 describes the simulation study for selecting the maximum spatial cluster size to compare MCS-P with existing performance measures. Section 4 presents the application of MCS-P in case data of measles in Henan, China, and Section 5 provides the discussion and conclusions.

## Methods

### Spatial scan statistic

Spatial scan statistics are used to identify the maximum likelihood clusters in the form of a set *Z* of spatial units, which reject the null hypothesis in the study area *G* and consider *p* and *q* as the probability of an event that occurs inside and outside a zone, respectively. In current applications, we usually focus on detecting zones where *p*>*q*.

Although spatial scan statistics vary in terms of the shape of scanning window and the probability model, most of them employ the logarithm of the likelihood ratio (LLR) as the test statistic to identify maximum likelihood clusters[17–22]. A maximum likelihood estimation method is also applied to determine the most clustered sub-region *Z*. The detected cluster $\hat{Z}$ is the maximum likelihood estimator of *Z*. Let *C* and *c*_*z*_ be the observed number of events in *G* and *z*, respectively, whereas *N* and *n*_*z*_ are the expected number of events in *G* and *z* under the null hypothesis; hence, *N* = *C*. Let *L*(*z*) be the likelihood under the alternative hypothesis that *z* is a cluster and *L*_0_ be the likelihood under the null hypothesis; in this case, LLR is:

$$\begin{array}{l} \frac{L(z)}{L_{0}}={\left(\frac{c_{z}}{n_{z}}\right)}^{c_{z}}{(\frac{C-c_{z}}{C-n_{z}})}^{C-c_{z}}\\ LLR(z)=\text{ln}\frac{L(z)}{L_{0}} \end{array}$$ (1)

where *L*_0_ is a constant for a given *G*. The collection *z* of spatial units can maximize *LLR*(*z*) and *L*(*z*).

A scanning window with a pre-defined shape and maximum spatial size is employed to identify the solution $\hat{Z}=\{Z|LLR(Z)\geq LLR(Z^{\prime })\forall Z^{\prime }\in G\}$. The size (*η*) of the window varies between zero and the maximum spatial cluster size (*η*(*Z*)) on each possible focus in *G* to generate a set of potential clusters: P = ∪{*z*|*η*(*z*) ≤ *η*(*Z*)}. The potential cluster in P that maximizes the likelihood is the estimator of *Z* and is also called the most likely cluster (MLC). In addition to this MLC, secondary clusters with high likelihood values are considered.

The precise distribution of the test statistic remains unclear; thus, a Monte Carlo simulation is employed to obtain the critical value under the null hypothesis. The LLRs of all potential clusters are compared with the critical value to determine their significant differences.

### Performance measures

Although the capacity to detect the presence of clusters has been widely studied[18, 22–27], the performance or the so-called spatial accuracy of the detected clusters should also be considered[14, 16, 20]. In most studies, measures concerning two respective aspects of performance are used in pairs[21, 28–30], with one measure exhibiting the capacity to correctly identify spatial units inside the true clusters and the other measure possessing the capacity to correctly identify spatial units outside the true clusters. Moreover, measures accounting for both aspects are used to measure the overall performance[5, 31]. The three commonly used performance measures include sensitivity, positive predictive value (PPV), and misclassification. Previous studies used the number of spatial units to calculate performance measures; however, the use of a population-based measure can provide more robust estimates[32]. Read et al.[14] stated that all spatial units in a study region can be classified into four types to evaluate the performance of the spatial scan statistic:

1. Units inside both true and detected cluster(s)
2. Units inside the true cluster(s) but outside the detected cluster(s)
3. Units inside the detected cluster(s) but outside the true cluster(s)
4. Units outside both true and detected cluster(s).

Let the population in each of the four types of spatial units be *a*, *b*, *c*, and *d*. The three common performance measures are described as follows:

$$\text{sensitivity}=\frac{a}{a+b}.$$ (2)

Sensitivity represents the proportion of the population in the true cluster(s) that is correctly identified as cluster(s). This measure is used to determine the capacity to determine true cluster(s).

$$\text{PPV}=\frac{a}{a+c}.$$ (3)

PPV, which is commonly used with sensitivity, represents the proportion of the population in the detected cluster(s) which actually belongs to the true cluster(s). This measure indicates the capacity to accurately identify spatial units outside the true cluster(s).

$$\text{misclassification}=\frac{b+c}{a+b+c+d}.$$ (4)

Misclassification represents the proportion of mistakenly identified populations. This measure accounts for the population of spatial units within the true cluster(s) but outside the detected cluster(s), as well as the population of detected spatial units outside the true cluster(s). If the misclassification is equal to zero, then all spatial units are correctly identified.

These performance measures are based on the given presence of true clusters and are not applicable for real data sets with unknown clusters. In this study, we propose a novel overall performance measure by using the applied data set.

### Novel overall performance measure based on applied data sets

Let MLC with *η*(*Z*) = *i* be ${\hat{Z}}_{i1}$ and the *j*th significant cluster be ${\hat{Z}}_{ij}$, then the maximum spatial cluster size *η*(*Z*) is a parameter of the collection of potential clusters P = ∪{*z*|*η*(*z*) ≤ *η*(*Z*)}. For a local optimum with *η*(*Z*) = *i*, ${\hat{Z}}_{i1}=\{Z|LLR(Z)\geq L(Z^{\prime })\forall Z^{\prime }\in {Ρ}_{i}\}$ may differ from the global optimum $\hat{Z}=\{Z|LLR(Z)\geq LLR(Z^{\prime })\forall Z^{\prime }\in G\}$. Therefore, $LLR({\hat{Z}}_{i1})$ may be smaller than $LLR(\hat{Z})$. When only MLC is found or of interest, the optimal *η*(*Z*) is selected by ranking the LLR of different MLCs. The optimal *η*(*Z*) = *i* maximizes $LLR({\hat{Z}}_{i1})$.

Comparison of the LLR of the corresponding clusters, such as MLC, may be insufficient for ranking the performance with different *η*(*Z*) values when secondary clusters are of interest. First, pairing of the corresponding multiple clusters is very complicated. Second, multiple outcomes from comparisons of different paired clusters may not be consistent. For instance, $LLR({\hat{Z}}_{i_{2}1})$ can be smaller than $LLR({\hat{Z}}_{i_{1}1})$, whereas $LLR({\hat{Z}}_{i_{2}2})$ can be larger than $LLR({\hat{Z}}_{i_{1}2})$. When all the significant clusters are of interest, the significant clusters classify all spatial units into two sets: clustering set in which events are likely to cluster, as well as the set in which events in the rest of the spatial units are not likely to cluster. Therefore, the union of all significant clusters, instead of individual clusters, can be used as the clustering set when ranking the performance of multiple clusters. Let the union of significant clusters found with *η*(*Z*) = *i* be *Z*_*i*0_, then

$$Z_{i0}=\cup_{j}Z_{ij}$$ (5)

Clustering sets with different *η*(*Z*) values can be used to rank the performance of multiple clusters in a manner similar to that of MLC. With *η*(*Z*) maximizing the likelihood under the alternative hypothesis, events in the clustering set are least likely to cluster by chance. Moreover, the likelihood function is maximized when LLR is maximized. Therefore, comparison of the LLR of the clustering set can rank the performance of multiple clusters with different *η*(*Z*) values.

$$LLR(Z_{i0})=\text{ln}{\left(\frac{c_{Z_{i0}}}{n_{Z_{i0}}}\right)}^{c_{Z_{i0}}}{\left(\frac{C-c_{Z_{i0}}}{C-n_{Z_{i0}}}\right)}^{C-c_{Z_{i0}}}$$ (6)

The LLR conditioned on *Z*_*i*0_ represents the ratio of the likelihood of the clustering set with *η*(*Z*) = *i* and the likelihood under the null hypothesis. LLR can also measure the dissimilarity between *Z*_*i*0_ as the clustering set and the null hypothesis. When *η*(*Z*) = *i* maximizes the *LLR*(*Z*_*i*0_), *L*(*Z*_*i*0_) will also be maximized. That is, when *η*(*Z*) = *i* maximizes *LLR*(*Z*_*i*0_), the events in Z_i0_ are more likely to cluster than any other clustering sets found with other *η*(*Z*) values.

Although *LLR*(*Z*_*i*0_) can be used to rank the performance of the spatial scan statistic with different *η*(*Z*) values, the range of *LLR*(*Z*_*i*0_) may differ because of the spatial distribution of events. For instance, a non-clustering spatial unit surrounded by clusters may be included in the scanning window. The non-clustering spatial units with relatively high RR near a cluster are more likely to be included in Z_i0_ than those far from the clusters. This trend causes varied ranges and optimal values of *LLR*(Z_i0_), even in data sets generated with the same model. In addition, most existing performance measures are built in the form of proportions and rahnge from 0 to 1. Thus, the adjustment of the effect of spatial distribution on the data set would render the measure comparable with existing performance measures. An approximate maximum of LLR from *G* is therefore used. The spatial scan statistic is employed to detect clustering spatial units with *p*>*q*; hence, the union of spatial units with RR higher than 1 is selected as the most clustering set (MCS) to obtain the approximate maximum LLR in *G*.

$$Z_{MCS}=\cup \left\{z|z\in G,p_{z}>q_{z}\right\}$$ (7)

Subsequently, we adjust *LLR*(*Z*_*i*0_) with *LLR*(*Z*_*MCS*_), such that the performance measure MCS-P is:

$$MCS-P=\frac{LLR(Z_{i0})}{LLR(Z_{MCS})}$$ (8)

MCS-P represents the ratio between the LLR of the clustering set with *η*(*Z*) = *i* and the approximate maximum LLR in *G*. LLR describes the relative support of the alternative hypothesis against the null hypothesis. MCS-P presents the closeness of the relative support of the clustering set to the maximum support obtained from the dataset. With this adjustment, MCS-P ranges from 0 to 1, which is similar to that of other performance measures. The denominator *LLR*_*MCS*_ is the approximate maximum LLR obtained from *G*. In extreme cases, the LLR of the clustering set may be higher than that of MCS. Although no such case was found in the present study, we should note that MCS-P is an approximate relative performance measure.

## Simulation Study

### Simulation data

Simulated benchmark data sets based on a real data set of breast cancer mortality [33] were used in this study. The population at risk in the simulated data analysis is the female population from the 1990 census, which contains 29,535,210 individuals in Northeastern USA. The study region consists of 245 counties in Northeastern USA[23].

Fifty scenarios were built with 50 different circular cluster models. The models contain two different total simulated case numbers of 600 or 6000; five different cluster sizes of 1, 2, 4, 8, or 16 counties; and five cluster spatial distribution patterns containing one cluster located in rural, mixed, or urban area. Two clusters were located in rural and urban areas, whereas three clusters were located in all the three areas. These benchmark datasets are available at the SatScan website[34] and commonly used to evaluate different clustering tests[23] or spatial scan statistics with scanning windows of different shapes and parameters[24, 32, 35]. Details of the cluster models are given in Table 1.

**Table 1:** Simulated cluster models.

Cluster size	Total simulated cases	600					6000				
	Cluster location	Rural	Mixed	Urban	Two	Three	Rural	Mixed	Urban	Two	Three
1	*E(c/H*_*A*_*)*	10	39	42	52	91	13	208	226	239	447
	*E(c/H*_*0*_*)*	0.05	14.43	15.97	16.02	30.45	0.5	144.3	159.7	160.2	304.5
	RR	192.89	2.85	2.73	3.24	2.99	23.73	1.45	1.43	1.51	1.51
	Population	2675	710196	786178	788853	1499049	2675	710196	786178	788853	1499049
2	*E(c/H*_*A*_*)*	12	42	50	62	104	23	231	293	316	547
	*E(c/H*_*0*_*)*	0.46	16.41	21.78	22.24	38.65	4.6	164.1	217.8	222.4	386.5
	RR	27.03	2.70	2.43	2.79	2.68	4.96	1.42	1.36	1.44	1.45
	Population	22911	817050	1072181	1095092	1912142	22911	817050	1072181	1095092	1912142
4	*E(c/H*_*A*_*)*	18	51	100	118	169	59	302	716	775	1077
	*E(c/H*_*0*_*)*	2.69	22.52	59.99	62.68	85.2	26.9	225.2	599.9	626.8	852
	RR	7.05	2.40	1.81	1.88	1.98	2.21	1.36	1.22	1.27	1.32
	Population	132343	1108440	2953077	3085420	4193860	132343	1108440	2953077	3085420	4193860
8	*E(c/H*_*A*_*)*	22	58	150	172	230	80	358	1162	1242	1600
	*E(c/H*_*0*_*)*	4.16	27.47	101.96	106.12	133.59	41.6	275.7	1019.6	1061.2	1336.9
	RR	5.35	2.24	1.63	1.62	1.72	1.92	1.32	1.17	1.21	1.27
	Population	204829	1352284	5018909	5223738	6576022	204829	1352284	5018909	5223738	6576022
16	*E(c/H*_*A*_*)*	28	67	209	237	304	121	434	1713	1834	2268
	*E(c/H*_*0*_*)*	7.32	34.22	154.94	162.26	196.48	73.2	342.2	1549.4	1622.6	1964.8
	RR	3.9	2.1	1.53	1.46	1.55	1.66	1.29	1.15	1.19	1.25
	Population	360275	1684327	7627173	7987448	9671775	360275	1684327	7627173	7987448	9671775

Note: *E(c/H*_*A*_*)* and *E(c/H*_*0*_*)* are the expected number of cases under the alternative and null hypotheses, respectively. RR is the relative risk.

In the latter part of the paper, scenarios are mentioned in the form of “total case numbers-cluster location-cluster size.” For instance, 600-two-1 refers to the data sets in the scenario with 600 total simulated cases and two clusters located in urban and rural areas, with each cluster covering only one county.

### Spatial scan parameters

Each of the 50 different maximum spatial cluster sizes were set to increase from 1% to 50% by increments of 1% for the total population at risk for each data set. Only clusters with P-values less than 0.05 were considered significant. In cases when no significant clusters were detected, the detected population was set to zero. Based on previous study, the inclusion of secondary clusters that overlap with more likely clusters does not improve the performance[5]. As the default reporting criteria for secondary clusters, only the secondary clusters unrelated to any likely clusters were reported. With each maximum spatial cluster size, the performance of the circular scan statistic was evaluated by MCS-P with three existing performance measures, such as sensitivity, PPV, and misclassification.

### Agreements of MCS-P with other performance measures in different scenarios

To validate MCS-P for different cluster models, we defined the values of each performance measure that differs by less than 0.01 (1%) for each data set as the values close to the optimal result[5]. The 50 different maximum spatial cluster sizes were classified into four types for each data set:

1. Results of MCS-P and the other performance measure are close to the optimal result
2. Only the result of the other performance measures is close to the optimal result
3. Only the result of MCS-P is close to the optimal result
4. None of the results of MCS-P and the other performance measure are close to the optimal result.

For types 1 and 4, MCS-P provided results similar to those of other performance measures. The agreement of MCS-P with each existing performance measure was reported for each cluster model. Agreement represents the similarity of MCS-P and other performance measures for identifying whether a maximum spatial cluster size is close to the optimal value. An agreement of 100% indicates that all 50 maximum spatial cluster sizes are accurately identified and similar between that derived from MCS-P and from another existing performance measure.

The average agreement values of MCS-P with sensitivity, PPV, and misclassification are 86.6257%, 66.2698%, and 81.3829%, respectively. Most MCS-P results are similar to those of sensitivity and misclassification. Although relatively low at more than 66%, the results obtained using MCS-P are similar to those of PPV. Generally, when the values of MCS-P are close to the optimal results, other performance measures also achieve values close to the optimal results. Moreover, MCS-P works better with sensitivity than that with misclassification and PPV. Despite the arbitrary cut-off points for determining whether the values of the performance measures are close to their optimal results, the results show the high agreement of MCS-P and the other performance measures.

The agreements of MCS-P in different scenarios are shown in Table 2. For 45 scenarios, MCS-P exhibits high agreement values with other performance measures similar to the average agreement. For five scenarios including 600-two-1, 600-two-2, 6000-two-1, 600-three-1, and 6000-three-1, MCS-P exhibits low agreements with the other performance measures.

**Table 2:** Agreements of MCS-P with the other performance measures in different scenarios.

	Cluster(s) locations	Rural area		Mixed area		Urban area		Rural and urban areas		Rural, mixed and urban areas	
Cluster sizes	Total simulated cases	600	6000	600	6000	600	6000	600	6000	600	6000
1	Sensitivity	0.9624	0.9678	0.9824	0.9658	0.9802	0.9810	0.2488	0.3426	0.4678	0.8440
	PPV	1.0000	0.9900	0.6728	0.7824	0.8828	0.8734	0.3328	0.4654	0.6826	0.4476
	Misclassification	1.0000	0.9836	0.8288	0.9034	0.9112	0.9386	0.2588	0.4078	0.6076	0.3904
2	Sensitivity	0.9558	0.9494	0.9470	0.9566	0.9862	0.9856	0.5266	0.6150	0.7908	0.8626
	PPV	0.9688	0.9900	0.7034	0.6758	0.9036	0.8668	0.5824	0.6572	0.4590	0.4658
	Misclassification	0.9664	0.9518	0.8620	0.8542	0.9066	0.8730	0.5508	0.6652	0.7552	0.8092
4	Sensitivity	0.9928	0.9704	0.9322	0.9244	0.9346	0.9082	0.7326	0.7850	0.8418	0.8626
	PPV	0.9706	0.9606	0.4798	0.5008	0.7498	0.7742	0.6762	0.7354	0.5296	0.5186
	Misclassification	0.9612	0.9328	0.8172	0.8316	0.8054	0.8182	0.7208	0.7756	0.8250	0.8338
8	Sensitivity	0.9928	0.9432	0.9298	0.9488	0.9418	0.9342	0.8192	0.7738	0.8622	0.8564
	PPV	0.9706	0.7952	0.3886	0.4826	0.6186	0.6016	0.5674	0.5958	0.6138	0.4810
	Misclassification	0.9612	0.8656	0.8130	0.8670	0.7584	0.7408	0.7580	0.6970	0.8190	0.8112
16	Sensitivity	0.9738	0.9306	0.8946	0.9540	0.9466	0.9472	0.8238	0.8796	0.8402	0.8950
	PPV	0.6316	0.7556	0.4442	0.4056	0.5532	0.5828	0.5114	0.5082	0.5834	0.5304
	Misclassification	0.9428	0.9096	0.8046	0.8594	0.7966	0.8318	0.7854	0.7960	0.8086	0.8958

Note: Scenarios with low agreements of MCS-P with other performance measures are underlined.

For measuring the capacity to accurately detect true clusters, the results of MCS-P are generally similar to sensitivity in all scenarios, except for the abovementioned five cases. For measuring the capacity for correct identification of spatial units outside the clusters, the agreement of MCS-P with PPV varies in different scenarios. In 600-rural-1, which generated the highest RR and the smallest population, MCS-P exhibits high agreement with PPV. For large clusters with low RR, the agreement of MCS-P with PPV decreases. As an overall performance measure, MCS-P manifests similar results to those of misclassification for most scenarios. That is, with PPV and misclassification, MCS-P is highly accurate in scenarios containing clusters with high RR and small populations. Moreover, MCS-P with sensitivity always exhibits high agreement in most scenarios.

The result of MCS-P with the other performance measures is less accurate in the five scenarios than that in the other scenarios; as such, multiple clusters are generated by different cluster models. One cluster is constructed with very high RR and a small population in a rural area, whereas other cluster(s) possess low RR with a large population in urban areas (and mixed areas), as shown in Table 1. Hence, the clusters are highly heterogeneous. Based on the likelihood of all clustering zones, MCS-P treats clustering zones from different clusters as a homogeneous clustering set. Therefore, in cases with multiple instances of highly heterogeneous clusters, larger values of MCS-P are more likely to be achieved when only the cluster with high RR and a small population is included compared with that when all the clusters are included. The clusters become less heterogeneous with increasing cluster sizes; therefore, the agreements of MCS-P with the other performance measures increase. Additional details are provided in the comparison of the average MCS-P and other measures for different cluster models.

### Comparison of average MCS-P and the other performance measures for each maximum spatial cluster size

In each scenario, the maximum spatial cluster sizes near the optimal value were selected with MCS-P. The values of other performance measures with the selected maximum spatial cluster sizes were compared with those of measures with other maximum spatial cluster sizes to determine whether the selection can improve the performance of the spatial scan statistic. The mean values of the performance measures over replicas in the same scenarios were reported for each maximum spatial cluster size to provide detailed information regarding the relationship between MCS-P and the other performance measures.

Generally, with the selected maximum spatial cluster sizes in most scenarios, high values of MCS-P correspond to high values of sensitivity and PPV and low values of misclassification. Selection of the maximum spatial cluster sizes using MCS-P, sensitivity, PPV, and misclassification suggests that the spatial scan statistics achieve accurate results for most cluster models.

The comparison of averaged MCS-P and other performance measures in different scenarios shows their detailed relationships. The average MCS-P is positively related to average sensitivity and PPV but negatively associated with average misclassification in most scenarios. Similar to the agreements of MCS-P with the other performance measures, the five scenarios containing highly heterogeneous clusters, namely, 600-two-1, 600-two-2, 6000-two-1, 600-three-1, and 6000-three-1, exhibit different relationships between MCS-P and the other performance measures.

**Fig 1:**
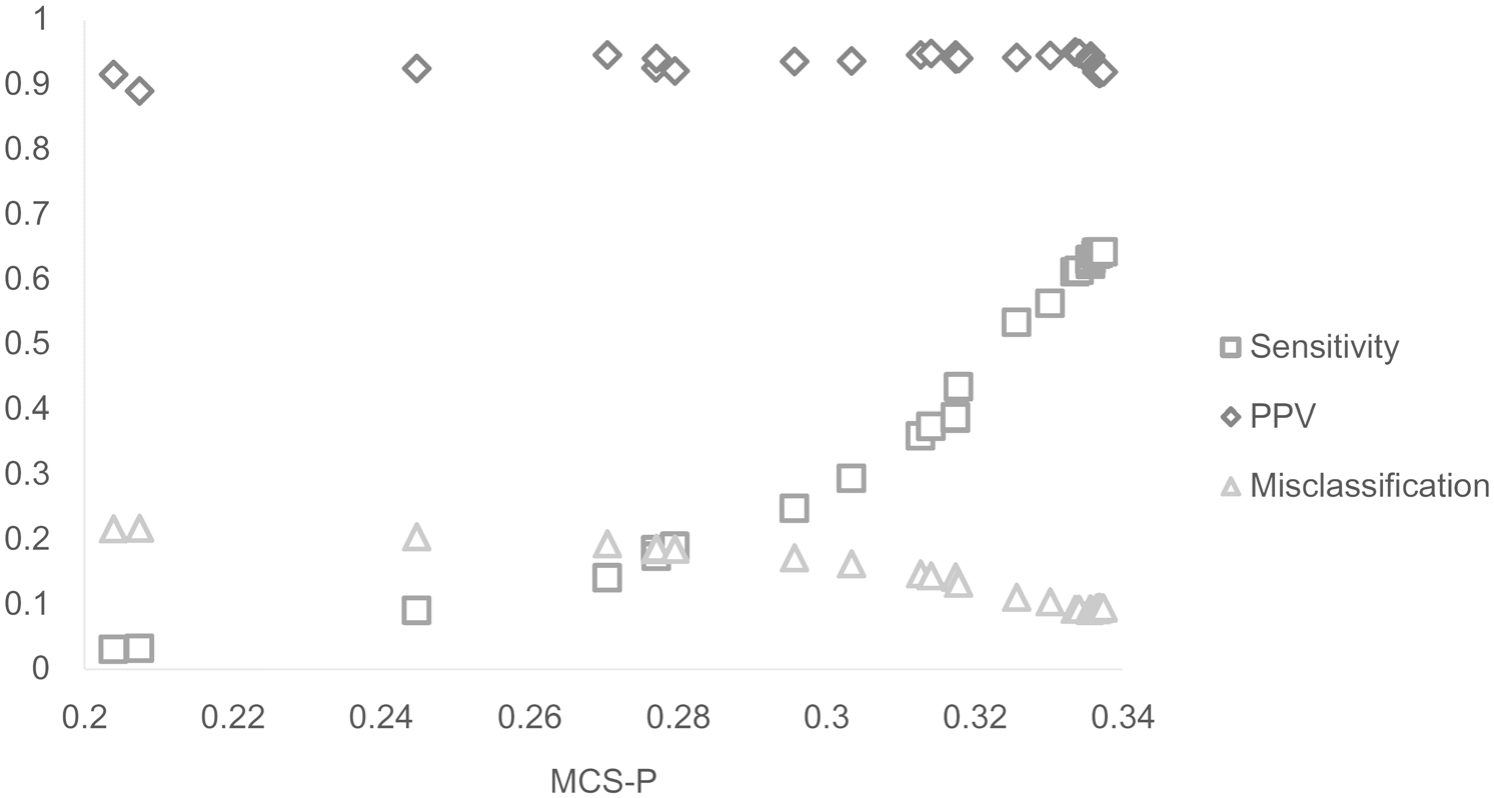
Average MCS-P and other measures in 6000-three-8.

The relationship between average MCS-P and the other performance measures are presented for several typical scenarios. The summary of 6000-three-8 (Table 3) shows the relationship between the average MCS-P and the other performance measures. The optimal results of each measure are marked in boldface, whereas values that differ by less than 0.01 (1%) from the optimal results are underlined. For the underlined values of MCS-P, the sensitivity and misclassification mostly overlap, which implies that the spatial scan statistic with the maximum spatial cluster sizes selected by MCS-P achieve values close to the optimal results of these measures. Therefore, maximum spatial cluster sizes can be selected with MCS-P to obtain accurate results for other performance measures. Hence, selection of the maximum spatial cluster sizes with MCS-P can improve the performance of spatial scan statistics. Detailed relationships between MCS-P and other performance measures are presented in Fig 1. The results of the other performance measures become closer to the optimal results with increasing MCS-P. Similar relationships between MCS-P and other performance measures can be found in the remaining 45 scenarios.

**Table 3:** Average performance measures for different maximum spatial cluster sizes in 6000-three-8.

Maximum spatial cluster size	MCS-P	Sensitivity	PPV	Misclassification
1	0.2039	0.0308	0.9157	0.2166
2	0.2074	0.0319	0.8905	0.2168
3	0.2448	0.0904	0.9251	0.2037
4	0.2705	0.1405	0.9458	0.1929
5	0.2771	0.1742	0.9258	0.1860
6	0.2770	0.1838	0.9406	0.1850
7	0.2796	0.1894	0.9215	0.1845
8	0.2957	0.2475	0.9356	0.1713
9	0.3034	0.2943	0.9363	0.1620
10	0.3127	0.3598	0.9462	0.1470
11	0.3141	0.3741	0.9478	0.1439
12	0.3174	0.3868	0.9456	0.1414
13	0.3174	0.3881	0.9399	0.1420
14	0.3178	0.4345	0.9401	0.1322
15	0.3256	0.5335	0.9423	0.1108
16	0.3301	0.5632	0.9448	0.1039
17	0.3335	0.6113	**0.9503**	0.0925
18	0.3341	0.6141	0.9480	0.0924
19	0.3356	0.6232	0.9341	0.0915
20	0.3356	0.6298	0.9411	**0.0910**
21	0.3355	0.6297	0.9444	0.0927
22	0.3357	0.6307	0.9345	0.0924
23	0.3363	0.6384	0.9300	0.0918
24	0.3367	0.6400	0.9193	0.0928
25	0.3369	**0.6423**	0.9254	0.0934
26	0.3371	**0.6423**	0.9177	0.0938
27	0.3372	**0.6423**	0.9177	0.0939
28	**0.3373**	**0.6423**	0.9172	0.0942
29	0.3364	0.6418	0.9215	0.0943
30	0.3368	0.6418	0.9205	0.0950
31	0.3368	0.6418	0.9201	0.0951
33–50	0.3368	0.6418	0.9193	0.0953

Note: Values with a distance less than 0.01 (1%) from the optimal values are underlined. Boldface values are the optimal results of each performance measure.

The five scenarios show the irregular relationship, similar to the agreements of MCS-P and the other performance measures. Given its conditioning on the likelihood of all significant clusters as a homogeneous clustering set, MCS-P cannot correctly measure the performance of multiple instances of highly heterogeneous clusters. This trend is particularly typical in 600-two-1, as shown in Table 4. One cluster in the rural area presents a high RR value of 192.89 and a very small population of 2675, whereas another cluster in the urban area has a large population of 786178 but a low RR value of 2.73. In the 600-two-1 scenario, the exclusive inclusion of the former cluster provides higher MCS-P values for the maximum spatial cluster sizes of 1% and 2% of the population at risk (Table 5). When parts or the entire latter cluster is included with a large maximum spatial cluster size of over 3%, MCS-P sharply decreases.

**Table 4:** Average performance measures for different maximum spatial cluster sizes in 600-two-1.

Maximum spatial cluster size	MCS-P	PPV	Sensitivity	Misclassification
1	0.5729	0.0034	0.9516	0.0268
2	**0.5758**	0.0034	0.9710	0.0267
3	0.4160	0.8704	**0.9887**	**0.0036**
4–7	0.4131	0.8804	0.9700	0.0040
8–50	0.4121	**0.8904**	0.9634	0.0042

Note: Values with a distance less than 0.01 (1%) from the optimal values are underlined. Boldface values are the optimal results of each performance measure.

**Table 5:** Average performance measures for different maximum spatial cluster sizes in 6000-two-16.

Maximum spatial cluster size	MCS-P	PPV	Sensitivity	Misclassification
1	0.1851	0.9634	0.0287	0.2630
2	0.2061	0.9190	0.0381	0.2613
3	0.2107	0.9191	0.0418	0.2603
4	0.2127	0.9157	0.0489	0.2587
5	0.2189	0.9298	0.0644	0.2546
6	0.2202	0.9307	0.0730	0.2523
7	0.2217	0.9327	0.0804	0.2503
8	0.2264	0.9349	0.1042	0.2442
9	0.2269	0.9384	0.1252	0.2385
10	0.2245	0.9399	0.1507	0.2321
11	0.2259	0.9317	0.1667	0.2288
12	0.2288	0.9381	0.1932	0.2213
13	0.2296	0.9372	0.2246	0.2132
14	0.2320	0.9320	0.2496	0.2075
15	0.2362	0.9354	0.2969	0.1952
16	0.2403	0.9401	0.3506	0.1804
17	0.2427	0.9451	0.3832	0.1713
18	0.2475	0.9464	0.4132	0.1635
19	0.2514	0.9475	0.4764	0.1468
20	0.2541	0.9472	0.4889	0.1436
21	0.2570	0.9516	0.5425	0.1290
22	0.2618	0.9532	0.5704	0.1212
23	0.2640	0.9603	0.6367	0.1027
24	0.2701	**0.9662**	0.6720	0.0933
25	0.2749	0.9640	0.7006	0.0868
26	0.2761	0.9595	0.7068	0.0863
27–28	0.2774	0.9567	0.7150	0.0850
29	0.2790	0.9457	0.7320	0.0839
30	0.2792	0.9425	0.7354	0.0843
31	**0.2795**	0.9363	0.7363	0.0862
32	0.2792	0.9348	0.7442	0.0853
33	0.2793	0.9321	0.7538	**0.0837**
34	0.2794	0.9311	0.7538	0.0841
35	0.2794	0.9312	0.7543	0.0840
36	0.2789	0.9276	0.7516	0.0862
37	0.2790	0.9277	0.7529	0.0860
38	0.2790	0.9249	0.7529	0.0870
39–40	0.2790	0.9254	0.7551	0.0866
41–50	0.2793	0.9238	**0.7647**	0.0855

Note: Values with a distance less than 0.01 (1%) from the optimal values are underlined. Boldface values are the optimal results of each performance measure.

This limitation disappears with increasing cluster sizes because of reduced heterogeneity of the clusters. If very small clusters with great heterogeneity are reported, then MCS-P may not be used as an appropriate performance measure.

**Fig 2:**
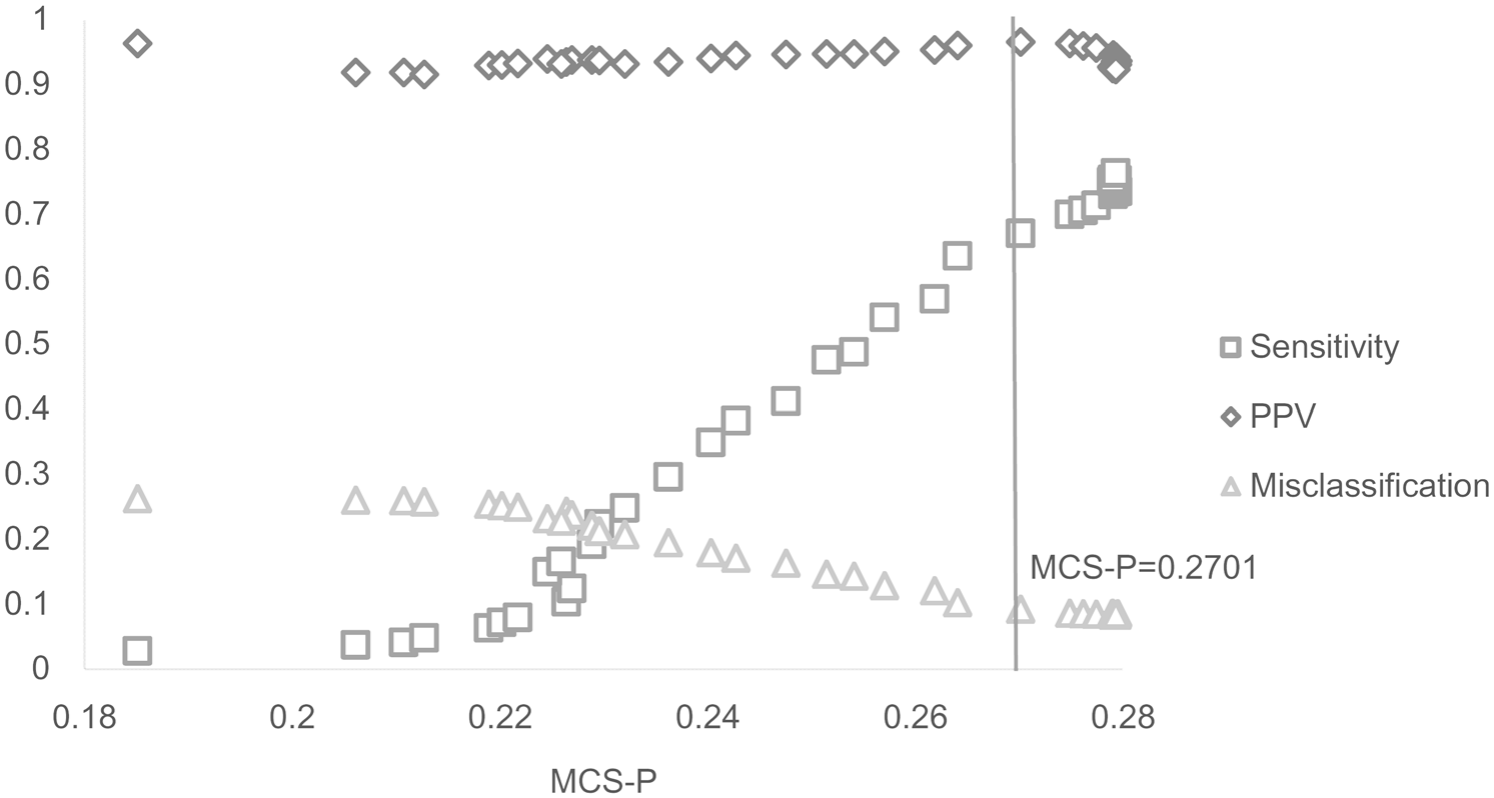
Average MCS-P and other measures in 6000-two-16. This figure shows two stages of the relationship between average MCS-P and the other performance measures. The vertical line shows the cut-off point where the first value close to the optimal results of MCS-P is achieved.

Interestingly, the statistic shows details of the relationships between MCS-P and the other performance measures. Results of 6000-two-16 describe clearly the details of this feature in Table 5 and Fig 2. The relationships between MCS-P and the three measures can be divided into two stages. The cut-off point is the very first value close to the optimal results of MCS-P, where MCS-P = 0.2701. During the first stage before MCS-P reaches the values close to the optimal results, sensitivity, PPV, and misclassification indicate that improved performance is achieved with increasing MCS-P. Hence, MCS-P works well with the three other performance measures. After MCS-P reaches the values close to the optimal results, other performance measures begin to fluctuate slightly around the optimal results. During this stage, MCS-P increases very slightly (no more than 0.01) when areas with relatively low RR and small populations are included. Under this condition, the values of the other measures may slightly decrease but are still close to the optimal results.

The fluctuation of existing performance measures around the optimal value of MCS-P is due to the fact that marginally expanding or reducing the cluster size will not significantly alter LLR[4]. Thus, MCS-P in such cases will slightly increase when including a new area with a relatively low RR but not a very large population. In these cases, other measures slightly decrease because the very small area should not be included. The inclusion of areas with plain RR and large population can decrease MCS-P. This trend can be found in 600-rural-1. In Table 6, the sensitivity remains equal to 1 when the true cluster is all included. However, large maximum spatial cluster sizes, including areas outside the true cluster, lead to a steep decrease in MCS-P. Therefore, the maximum spatial cluster sizes with MCS-P values close to the optimal results can be selected to improve the performance of the statistic.

**Table 6:** Average performance measures for different maximum spatial cluster sizes in 600-rural-1.

Maximum spatial cluster size	MCS-P	PPV	Sensitivity	Misclassification
1–2	**0.7599**	**0.9807**	**1**	**0.000081114**
3	0.7540	0.970734	**1**	0.000365707
4	0.7504	0.960753	**1**	0.000808159
5	0.7432	0.940789	**1**	0.001796976
6	0.7404	0.9308	**1**	0.002504661
7–50	0.7447	0.940779	**1**	0.002096859

Note: Values with distance less than 0.01 (1%) from the optimal values are underlined. Boldface values are the optimal results of each performance measure.

## Measles Incidence Data in Henan, China

We applied MCS-P to case data of measles on the county level in Henan province, China in May 2009; data were extracted from the disease reporting system of China CDC. A total of 1,371 cases of measles among a population of 91,669,661 were reported, and the annual incidence rate was 17.6 per 100,000. The data were analyzed using 50 maximum spatial cluster sizes following the simulation study. MCS-P was used for evaluation, and the result demonstrating the maximum MCS-P value was selected and compared with the result obtained using the default maximum spatial cluster size of 50% population at risk.

**Fig 3:**
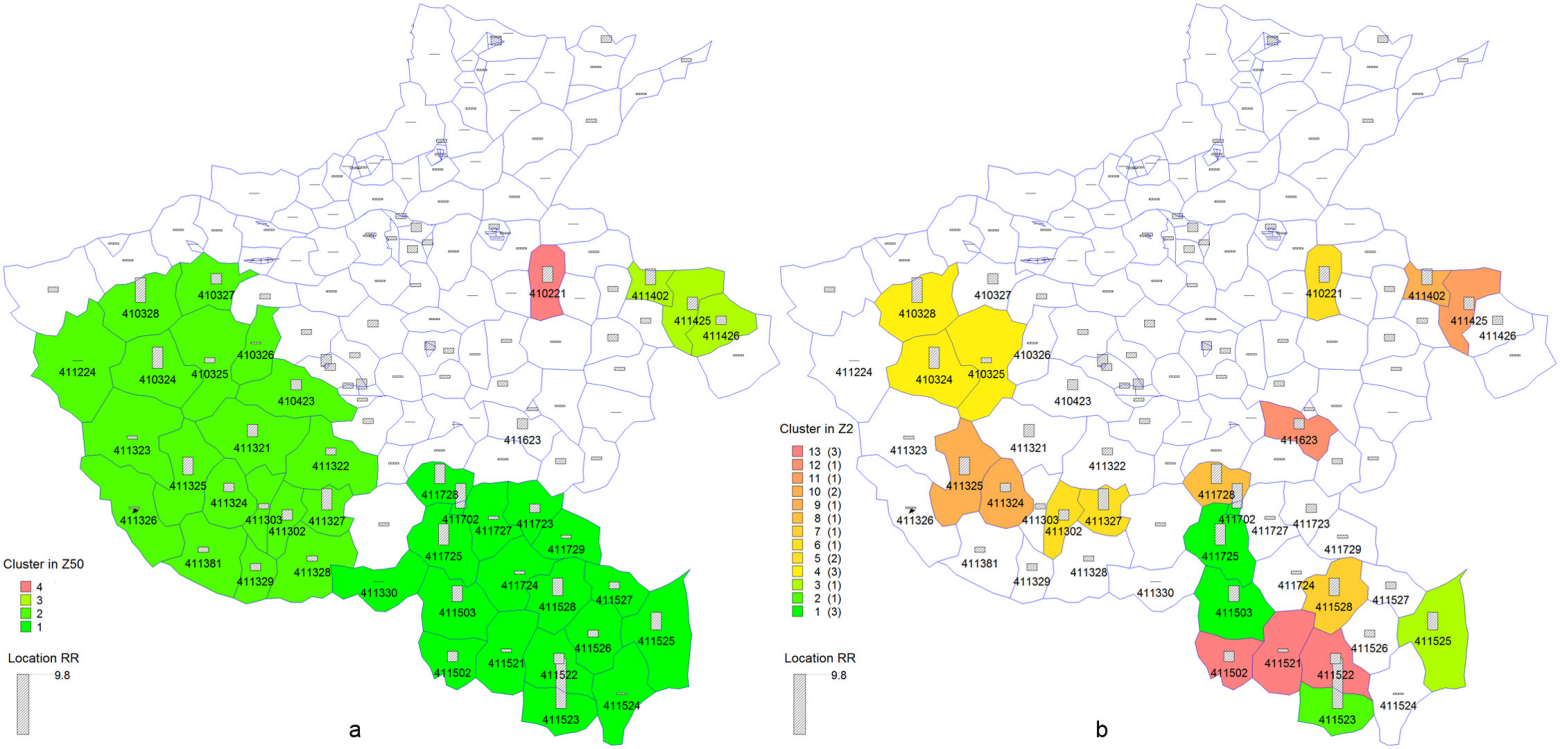
Incidence of measles in Henan in May 2009 and clusters detected with default (a) and selected maximum spatial cluster size using MCS-P (b). Administrative codes of 42 clustering counties are labeled. RR of each counties are presented as bar.

Maximum MCS-P was achieved when the maximum spatial cluster size was set to 2% of the total population. A total of 649 cases of 14,369,140 individuals in 21 counties were detected using a maximum spatial cluster size of 2% (Z2), whereas 886 cases of 28,859,679 in 41 counties were detected using a maximum spatial cluster size of 50% (Z50). The relative risk of Z2 and Z50 were 3.0200 and 2.0527, respectively, and both were located in the same parts of the study region. Variations in edges were found in clusters located southwest and southeast (Fig 3). To provide additional details, we compared the counties in Z2 and Z50. Twenty of 21 counties in Z2 were also found in Z50, and the remaining 22 counties that differed between Z2 and Z50 are shown in Table 7.

**Table 7:** Different counties in clusters detected using maximum spatial cluster sizes of 2% and 50%.

Counties	Cases	Population	RR	Cluster in Z2	Cluster in Z50
**411224**	**0**	**368491**	**0**	**n/a**	**2**
**411330**	**0**	**420199**	**0**	**n/a**	**1**
411326	2	665822	0.199677035	n/a	2
410326	2	406337	0.328122839	n/a	2
411524	4	570738	0.467054897	n/a	1
411729	8	955639	0.557153276	n/a	1
411727	9	799313	0.751226184	n/a	1
411323	5	420871	0.793592249	n/a	2
411724	9	709589	0.847050538	n/a	1
411381	19	1336473	0.949869751	n/a	2
411328	17	1193603	0.95170867	n/a	2
411303	12	839940	0.954863968	n/a	2
411329	12	642526	1.250955777	n/a	2
411526	14	692849	1.354691194	n/a	1
411322	20	913187	1.471270547	n/a	2
411527	13	580652	1.501736143	n/a	1
**411723**	**24**	**870989**	**1.857421518**	**n/a**	**1**
**411426**	**30**	**1062539**	**1.907699356**	**n/a**	**3**
410327	20	677480	1.988301511	n/a	2
**411623**	**32**	**1082690**	**1.999544224**	**12**	**n/a**
410423	26	859244	2.043008744	n/a	2
411321	23	586748	2.648641855	n/a	2

Counties in boldface are mentioned as examples.

A total of 12 of 22 counties showed lower incidence rates than the average value. Based on the purpose of scan statistics, these counties were probably incorrectly identified as clustering counties when searching for significantly high-risk spatial units. As shown in Fig 3, these counties, which were located at the edges of clusters 1 and 2 in Z50, are close to the high-risk counties. Therefore, these counties were included with near multiple high-risk areas in Z50. For instance, despite the absence of cases in 411224 and 411330, these counties were misclassified into Z50. In Z2, the use of MCS-P in maximum spatial cluster size selection excluded counties with low incidence rates.

Insignificant clustering counties with RR higher than 1 in Z50 were also excluded in Z2. These counties were surrounded by low-risk counties and were insignificant as a single potential cluster. In particular, 411723 showed higher RR (1.8574) than that of the surrounding counties and functioned as a potential cluster. The result suggested that high RR possibly occurred by chance. These counties were excluded in Z2 when MCS-P was used in the maximum spatial cluster size selection.

Although this county was insignificant in Z50, 411623 was regarded as the only clustering county in Z2 because the critical value of LLR is related to the maximum spatial cluster size. At a significance level of 0.05, the critical values for maximum spatial cluster size of 2% and 50% were 6.0221 and 7.5736, respectively. Therefore, 411623 (LLR = 6.0830) was significant in Z2 but not in Z50. When using MCS-P for maximum spatial cluster size selection, scan statistic is more sensitive to small clusters, whose ntest statistics are close to the critical value.

Moreover, 411426 was the only missed high-risk county contiguous to significant clusters in Z2. Three contiguous counties in the eastern region, such as 411426, 411425, and 411402, were tested to be a significant cluster in Z50. In Z2, the scanning window at a maximum spatial cluster size of 2% is too small to cover the three high-risk counties in the eastern region. Being one of the counties showing the lowest RR and is least likely to cluster out of the three counties, 411426 was tested as insignificant.

To sum up, the use of MCS-P in maximum spatial cluster size selection excluded counties with low incidence rates and insignificant high rates; these counties were incorrectly included in large clusters when using the default parameter. In addition, the former approach improved the capacity to identify small clusters showing relatively high incidence rates, which can be missed when using large critical value of test statistic with default maximum spatial cluster size. Although smaller maximum spatial cluster size may exclude a part of clustering areas, this phenomenon only occurs to the least likely clustering part of a cluster.

## Discussion

Spatial scan statistics are widely used in different fields to identify unusual clustering events throughout the study region. The maximum spatial cluster size is the only parameter of the circular scan statistic that affects its performance. Consistent with previous study, the present simulation study showed that the optimal maximum spatial cluster sizes vary in different scenarios[5]. As such, selection of a proper maximum spatial cluster size for each data set can improve the performance of the statistic because the cluster models of the practical data set are usually unknown. However, existing performance measures are inapplicable in most practical applications without known clusters. This limitation is addressed using the proposed MCS-P performance measure (Section 2.3). MCS-P is based on the likelihood of reported clusters and the approximate maximum likelihood from the used data set; therefore, this measure can be calculated without using the known presence of clusters. The simulation study also showed that the results of MCS-P are similar to those of other performance measures, namely, sensitivity, PPV, and misclassification, in most situations. Although MCS-P is not applicable for conditions with multiple highly heterogeneous clusters, this statistic can be used in most fields where clusters of interest exhibit similar characteristics. For instance, in epidemiological studies, outbreaks of the same disease usually share similar patterns for the same route of transmission, pathogen, and population at risk. In addition, customizing MCS-P for multiple highly heterogeneous clusters could be a direction for our future work.

The results of simulation study are conditioned to the data sets. Although the cluster models vary in terms of cluster number, location, RR level, and cluster size, this study presents several limitations. For example, in the benchmark data, the clusters generated are very far from each other such that no detected clusters can cover the parts of different clusters even when the maximum spatial cluster size is set to 50% population at risk. This phenomenon explains why the results do not differ in cases with large maximum spatial cluster sizes. However, the clusters may be located close to each other in actual practice as shown in the case data of measles. The detected clusters may include risky areas, which are not contiguous, and incorrectly include non-clustering areas around them. To address this problem, researchers should select parameters with MCS-P and alter the cluster shape, which would be investigated in our future work.

Two aspects of performance are considered to evaluate the spatial scan statistic. However, measures for detecting areas inside clusters may provide inverse evaluations against measures representing the capacity to identify areas outside the clusters. These results are common in simulation studies. Although the measures are conditioned to the data sets, the performance measures accounting for one specific aspect of performance, such as sensitivity and PPV, are likely to choose the largest or smallest maximum spatial cluster size. A large scanning window that reasonably covers more parts of the study region is more likely to contain the true clusters, whereas a small scanning window is less likely to contain areas outside the true clusters. Therefore, when focusing on sensitivity, large maximum spatial cluster sizes should be selected. By contrast, smaller maximum spatial cluster sizes should be selected when considering capacity to identify areas outside the clusters. In addition, the large maximum spatial cluster sizes should be selected first to avoid missing clusters. However, the overall performance is of more interest for most cases; as such, the preferred maximum spatial cluster size varies between different cluster models, and spatial scan statistics with different maximum spatial cluster sizes should be applied. Overall performance measures, such as misclassification and MCS-P, should also be used to select the maximum spatial cluster sizes to improve the performance. For data sets without true clusters, MCS-P may be the only performance measure that can be used. Before using MCS-P, the reported clusters still need to be checked. As shown in the simulation study, MCS-P may not work as an appropriate overall performance measure in the reported highly heterogeneous clusters. Although the selection of the maximum spatial cluster size with MCS-P can improve the performance of the statistic, this approach will consume more time than simply using the default setting. For instance, MCS-P has to be calculated for all the 50 maximum spatial cluster sizes to select the optimal maximum spatial cluster sizes. This step would consume as much as 50 times the original computation time. The computation time becomes longer as the applied data set becomes more complicated. Therefore, selection of a proper number of potential maximum spatial cluster size is important.

MCS-P is a measure based on significant clusters and thus varies among different significance thresholds. For example, the spatial scan statistic with parameters set to A may have higher values of MCS-P than those with parameter B at a significance threshold of 0.05 but may be lower at 0.01. Therefore, the significance threshold should be at the same level when comparing different detected values.

In conclusion, the results of using MCS-P in the simulation study are similar to those of three existing performance measures, namely, sensitivity, PPV, and misclassification, in most situations, except those with high heterogeneous clusters. MCS-P can be calculated without known true clusters and is therefore considered applicable to data sets without any given true clusters. The selection of the maximum spatial cluster size using MCS-P is helpful to achieve accurate results. Comparison of the average MCS-P and the other performance measures indicates that the selection of the maximum spatial cluster sizes with values close to the optimal results of MCS-P is a vital step to achieve satisfactory performance of the statistic.

## Supporting Information

S1 Appendix Evaluations of different maximum spatial cluster sizes from different performance measures for different cluster models. (XLS)

S2 Appendix Centroids coordinates, population, case data of measles in Henan Province, China in May, 2009. (XLS)

## References

[1] Kulldorff M. A spatial scan statistic. Communications in Statistics—Theory and Methods. 1997;26(6):1481–96. 10.1080/03610929708831995

[2] Kulldorff M. SaTScan-Software for the spatial, temporal, and space-time scan statistics. Boston: Harvard Medical School and Harvard Pilgrim Health Care 2010.

[3] Kulldorff M. Selected Applications by Field of Study [cited 2015 Feb 21]. Available from: http://www.satscan.org/references.html#Selected%20Applications%20by%20Field%20of%20Study.

[4] Kulldorff M. SaTScanTM User Guide for version 9.32014.

[5] Ribeiro SH, Costa MA. Optimal selection of the spatial scan parameters for cluster detection: a simulation study. Spatial and spatio-temporal epidemiology. 2012;3(2):107–20. Epub 2012/06/12. 10.1016/j.sste.2012.04.004 . 22682437

[6] Kulldorff M, Nagarwalla N. Spatial disease clusters: detection and inference. Statistics in medicine. 1995;14(8):799–810. Epub 1995/04/30. . 7644860 10.1002/sim.4780140809

[7] Forand SP, Talbot TO, Druschel C, Cross PK. Data quality and the spatial analysis of disease rates: congenital malformations in New York State. Health & Place Health & Place. 2002;8(3):191–9. 12135642 10.1016/s1353-8292(01)00037-5

[8] Coulston JW, Riitters KH. Geographic analysis of forest health indicators using spatial scan statistics. Environmental management. 2003;31(6):764–73. . 14565696 10.1007/s00267-002-0023-9

[9] Donnan PT, Parratt JD, Wilson SV, Forbes RB, O'Riordan JI, Swingler RJ. Multiple sclerosis in Tayside, Scotland: detection of clusters using a spatial scan statistic. Multiple sclerosis. 2005;11(4):403–8. . 16042222 10.1191/1352458505ms1191oa

[10] Chaput EK, Meek JI, Heimer R. Spatial analysis of human granulocytic ehrlichiosis near Lyme, Connecticut. Emerging infectious diseases. 2002;8(9):943–8. 10.3201/eid0809.020103 12194771 PMC2732548

[11] Weisent J, Rohrbach B, Dunn JR, Odoi A. Detection of high risk campylobacteriosis clusters at three geographic levels. Geospatial Health. 2011;6(1):65–76. WOS:000301177200009. 22109864 10.4081/gh.2011.158

[12] Marek L, Tuček P, Pászto V. Using geovisual analytics in Google Earth to understand disease distribution: a case study of campylobacteriosis in the Czech Republic (2008–2012). International journal of health geographics. 2015;14(1):1–13. 25628063 10.1186/1476-072X-14-7 PMC4328415

[13] Glaz J, Pozdnyakov V, Wallenstein S. Scan statistics methods and applications Boston: Birkhaäuser; 2009 Available from: 10.1007/978-0-8176-4749-0.

[14] complicatedRead S, Bath P, Willett P, Maheswaran R. Measuring the spatial accuracy of the spatial scan statistic. Spatial and spatio-temporal epidemiology. 2011;2(2):69–78. 10.1016/j.sste.2011.01.002 . 22749586

[15] Read S, Bath PA, Willett P, Maheswaran R. New developments in the spatial scan statistic. J Inf Sci Journal of Information Science. 2013;39(1):36–47.

[16] Huang L, Pickle LW, Das B. Evaluating spatial methods for investigating global clustering and cluster detection of cancer cases. SIM Statistics in Medicine. 2008;27(25):5111–42. 10.1002/sim.3342 PMC2575694 18712778

[17] Bhatt V, Tiwari N. A spatial scan statistic for survival data based on Weibull distribution. Statistics in medicine. 2014;33(11):1867–76. Epub 2013/12/20. 10.1002/sim.6075 . 24353112

[18] Tango T, Takahashi K. A flexible spatial scan statistic with a restricted likelihood ratio for detecting disease clusters. Statistics in medicine. 2012;31(30):4207–18. Epub 2012/07/19. 10.1002/sim.5478 . 22807146

[19] Jung I, Lee H. Spatial cluster detection for ordinal outcome data. Statistics in medicine. 2012;31(29):4040–8. Epub 2012/07/19. 10.1002/sim.5475 . 22807106

[20] Li XZ, Wang JF, Yang WZ, Li ZJ, Lai SJ. A spatial scan statistic for multiple clusters. Mathematical biosciences. 2011;233(2):135–42. Epub 2011/08/11. 10.1016/j.mbs.2011.07.004 . 21827771

[21] Jung I, Kulldorff M, Richard OJ. A spatial scan statistic for multinomial data. Statistics in medicine. 2010;29(18):1910–8. Epub 2010/08/04. 10.1002/sim.3951 20680984 PMC4147837

[22] Tango T, Takahashi K. A flexibly shaped spatial scan statistic for detecting clusters. International journal of health geographics. 2005;4:11 Epub 2005/05/21. 10.1186/1476-072x-4-11 15904524 PMC1173134

[23] Kulldorff M, Tango T, Park PJ. Power comparisons for disease clustering tests. Computational Statistics & Data Analysis. 2003;42(4):665–84. 10.1016/s0167-9473(02)00160-3

[24] Duczmal L, Kulldorff M, Lan H. Evaluation of Spatial Scan Statistics for Irregularly Shaped Clusters. Journal of Computational & Graphical Statistics. 2006;15(2).

[25] Torabi M, Rosychuk RJ. An examination of five spatial disease clustering methodologies for the identification of childhood cancer clusters in Alberta, Canada. Spatial and spatio-temporal epidemiology. 2011;2(4):321–30. 10.1016/j.sste.2011.10.003 . 22748230

[26] Ozonoff A, Bonetti M, Forsberg L, Pagano M. Power comparisons for an improved disease clustering test. Computational statistics & data analysis. 2005;48(4):679–84.

[27] Savory DJ, Cox KL, Emch M, Alemi F, Pattie DC. Enhancing spatial detection accuracy for syndromic surveillance with street level incidence data. International journal of health geographics. 2010;9:1 Epub 2010/01/20. 10.1186/1476-072x-9-1 20082711 PMC2819064

[28] Huang L, Kulldorff M, Gregorio D. A spatial scan statistic for survival data. Biometrics. 2007;63(1):109–18. Epub 2007/04/24. 10.1111/j.1541-0420.2006.00661.x . 17447935

[29] Jacquez GM. Cluster morphology analysis. Spatial and spatio-temporal epidemiology. 2009;1(1):19–29. 10.1016/j.sste.2009.08.002 20234799 PMC2838429

[30] Jung I, Kulldorff M, Klassen AC. A spatial scan statistic for ordinal data. Statistics in medicine. 2007;26(7):1594–607. Epub 2006/06/24. 10.1002/sim.2607 . 16795130

[31] Neill DB. An empirical comparison of spatial scan statistics for outbreak detection. International journal of health geographics. 2009;8:20 Epub 2009/04/18. 10.1186/1476-072x-8-20 19371431 PMC2691403

[32] Costa MA, Assunção RM, Kulldorff M. Constrained spanning tree algorithms for irregularly-shaped spatial clustering. Computational Statistics & Data Analysis. 2012;56(6):1771–83.

[33] Kulldorff M, Feuer EJ, Miller BA, Freedma LS. Breast cancer clusters in the northeast United States: a geographic analysis. American journal of epidemiology. 1997;146(2):161–70. 9230778 10.1093/oxfordjournals.aje.a009247

[34] Kulldorff M. Northeastern USA Benchmark Data, Purely Spatial 2015 [cited 2015 Feb 21]. Available from: http://www.satscan.org/datasets/nebenchmark/index.html.

[35] Kulldorff M, Huang L, Pickle L, Duczmal L. An elliptic spatial scan statistic. Statistics in medicine. 2006;25(22):3929–43. Epub 2006/01/26. 10.1002/sim.2490 . 16435334

